# Anthropogenic Disturbance Shapes Native–Alien Community Patterns Across Urban–Rural Gradients in a Neglected Invertebrate Model System

**DOI:** 10.1002/ece3.74369

**Published:** 2026-09-22

**Authors:** Alessandro Nota, Thomas Hesselberg

**Affiliations:** ^1^ Department for Continuing Education University of Oxford Oxford UK; ^2^ Department of Veterinary Sciences University of Turin Grugliasco Italy; ^3^ Department of Biology University of Oxford Oxford UK

**Keywords:** alien species, gastropods, human disturbance, land snails, Oxfordshire

## Abstract

Land snails represent a diverse yet understudied group of organisms, with new species continuously being described. However, numerous extinctions have recently occurred, largely due to the introduction of alien counterparts. Urbanisation and habitat disturbance are well‐known to promote the spread of opportunistic and alien species, but their effect on alien land snails has seldom been investigated. This research aimed to test whether alien snails' presence and abundance can be influenced by proximity to roads, water sources and human structures, as well as the site size and the presence of native species. Snail populations were surveyed in eight woodland sites across Oxfordshire, U.K., using systematic quadrat sampling. Species identification was based on morphological characteristics, complemented by molecular analyses for taxonomically ambiguous taxa. A total of 24 species (grouped in 23 taxa) were identified, including five aliens. The results indicate that alien snails are more prevalent in smaller and disturbed areas, with their abundance decreasing with increasing distance from roads. The presence of native species appears to exert biotic resistance against invasions, particularly in larger and less disturbed sites. These results highlight the critical role of human activities in shaping land snail communities, evidencing that roadsides can act as important corridors for alien species dispersal. In contrast, larger rural sites, with richer native communities, provide biological resistance against invasions. Given the growing trends of urbanisation and habitat alteration, future conservation strategies should focus on mitigating anthropogenic disturbances and preserving larger, interconnected natural areas to support native biodiversity and limit biological invasions.

## Graphical abstract

Urbanisation and habitat disturbance are well‐known to promote the spread of opportunistic and alien species, but their effect on alien land snails has seldom been investigated. This research aimed to test whether alien snails presence and abundance can be influenced by proximity to roads, water sources and human structures, as well as the site size and the presence of native species across Oxfordshire, UK. The results indicate that alien snails are more prevalent in smaller and disturbed areas, with their abundance decreasing with increasing distance from roads with native species appearing to exert biotic resistance against invasions.

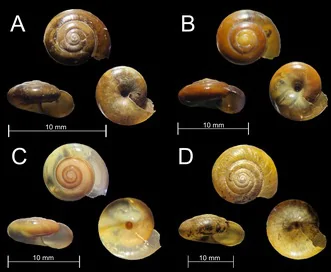

## 1 Introduction

Molluscs are one of the largest and most diverse phyla of invertebrates, encompassing a wide range of animals with different evolutionary histories, morphological structures, life cycles and ecological roles (Wanninger and Wollesen 2019). These organisms have adapted to nearly every environment on Earth, colonising marine, freshwater and terrestrial ecosystems (Rosenberg 2014). Terrestrial molluscs (snails and slugs) belong to the class Gastropoda and are classified into three distinct subclasses: Neritimorpha (4 families), Caenogastropoda (13 families) and Heterobranchia (120 families) (Proios et al. 2021; Molluscabase 2024). Many land snails are small and inconspicuous, often occurring within leaf litter, soil, moss, dead wood or other humid microhabitats, making it difficult to detect them without targeted sampling (Cameron 2008; Durkan et al. 2013). Together with the taxonomic expertise required for reliable identification and the broader research bias affecting less charismatic invertebrate groups, this may contribute to their frequent underrepresentation in ecological studies (Cardoso et al. 2011; Troudet et al. 2017). Nevertheless, land snails exhibit a huge global diversity, with nearly 25,000 recognised species, with new species constantly being described (Rosenberg 2014). From a conservation perspective, the extinction of numerous mollusc species has been documented in the last few centuries, with nonmarine molluscs accounting for 99% of the total molluscan extinctions (Lydeard et al. 2004). Introduced molluscs and other alien species have been repeatedly identified as important drivers of native mollusc declines and extinctions. This happens through various mechanisms, including predation, competition, fouling, hybridization and habitat alteration (Hadfield et al. 1993; Strayer 1999; Lydeard et al. 2004; Régnier et al. 2009; Curry and Yeung 2013; Williamson et al. 2021). Moreover, while alien species may sometimes occupy habitats or niches already altered by anthropogenic disturbance, this does not necessarily imply functional replacement of native taxa. In molluscs, introduced species can differ markedly from threatened or extinct native species in taxonomy, trophic interactions, and habitat use, so their establishment may further contribute to biotic homogenisation rather than restoring lost biodiversity (Strayer 1999; Karatayev et al. 2018). This issue is also scale‐dependent, since local increases in species richness due to alien establishment may mask losses of native diversity at broader spatial scales (Karatayev et al. 2018). However, despite the many ecological roles that molluscs play, the interactions between native and alien species have mainly been explored with other groups of organisms, while land snails, in particular, remain poorly studied in most regions of the world, leading to a potential underestimation of their ecological relevance. Studying how terrestrial gastropod communities are shaped by native–alien species interactions is therefore essential, given the numerous impacts alien molluscs can have on biodiversity, agriculture and even human health (Odendaal et al. 2008; Nurinsiyah and Hausdorf 2019).

Urbanisation and human disturbance are considered two of the main factors promoting the spread of opportunistic and alien species: in general, disturbance can create ‘open niches’ for invasion (Spyra et al. 2015), and therefore, human‐altered environments are often occupied by generalist species (Devictor et al. 2008), many of which are alien (Brasher 2003; Marvier et al. 2004; Spyra et al. 2015). This process is particularly evident in fragmented urban landscapes, where small and isolated reserves can act as refuges but are strongly exposed to edge effects and propagule pressure from surrounding human structures, tending to host more generalist and alien species. In contrast, larger and less fragmented reserves may reduce edge effects and support more stable native communities (McKinney 2002; Dudley et al. 2024). Such communities may, in turn, contribute to biotic resistance against invaders, although relationships between native and alien richness can be scale‐dependent and context‐specific (Beaury et al. 2020). Understanding how variables such as site size, proximity to roads and human structures interact in shaping snail communities can provide valuable estimates of the impacts caused by habitat fragmentation and loss.

Terrestrial gastropods are colloquially divided into two non‐monophyletic groups: slugs and snails. Slugs have a reduced and usually internal shell, while snails retain a clearly visible shell into which they can withdraw. Snails can usually be identified by the shell morphology and colour, while slug identification typically requires a body dissection to examine the reproductive organs (Cameron 2008; Rowson et al. 2014). In Britain, the collection, study and use of shells have been very popular for centuries, both for scientific research and recreational purposes (Gregory 2002; Oliver et al. 2020; Duggins 2023). The United Kingdom hosts around 100 species of terrestrial land snails (including semi‐slugs), but a large proportion (~20%) is believed to be introduced by humans (Cameron 2008). Cameron (2008) categorises the UK land snail fauna into five main groups: (i) a forest fauna: species moderately resistant to disturbance and surviving in ancient woodlands, constituting the largest group; (ii) a wetland fauna: species highly susceptible to anthropogenic pressures, including many rare and endangered taxa; (iii) a rock‐dwelling fauna: associated with limestone, and very vulnerable to airborne pollution; (iv) a sand dunes and dry calcareous grassland fauna: mainly composed of alien species; (v) a fauna of highly disturbed places: includes species in parks and gardens, i.e., both native woodland species that managed to adapt to this type of habitat and alien species.

This research aims to investigate the diversity of native and alien land snails in woodland habitats of Oxfordshire, U.K., focusing on the forested and highly disturbed habitat categories (i and v) described by Cameron (2008). Moreover, we hypothesised that the presence of alien snails would increase with anthropogenic disturbance, and thus be higher in urban and disturbed areas. Therefore, we tested whether alien species richness and abundance increase in smaller woodland reserves and in quadrats closer to roads and human infrastructures, and whether larger sites with richer native communities show lower levels of invasion. We also explored the potential role of proximity to water sources, given the ecological importance of humidity for terrestrial gastropods. With these analyses, we aim to clarify the factors influencing the spread and establishment of alien snails in such ecosystems.

## 2 Materials and Methods

### 2.1 Survey Period and Location

**FIGURE 1:**
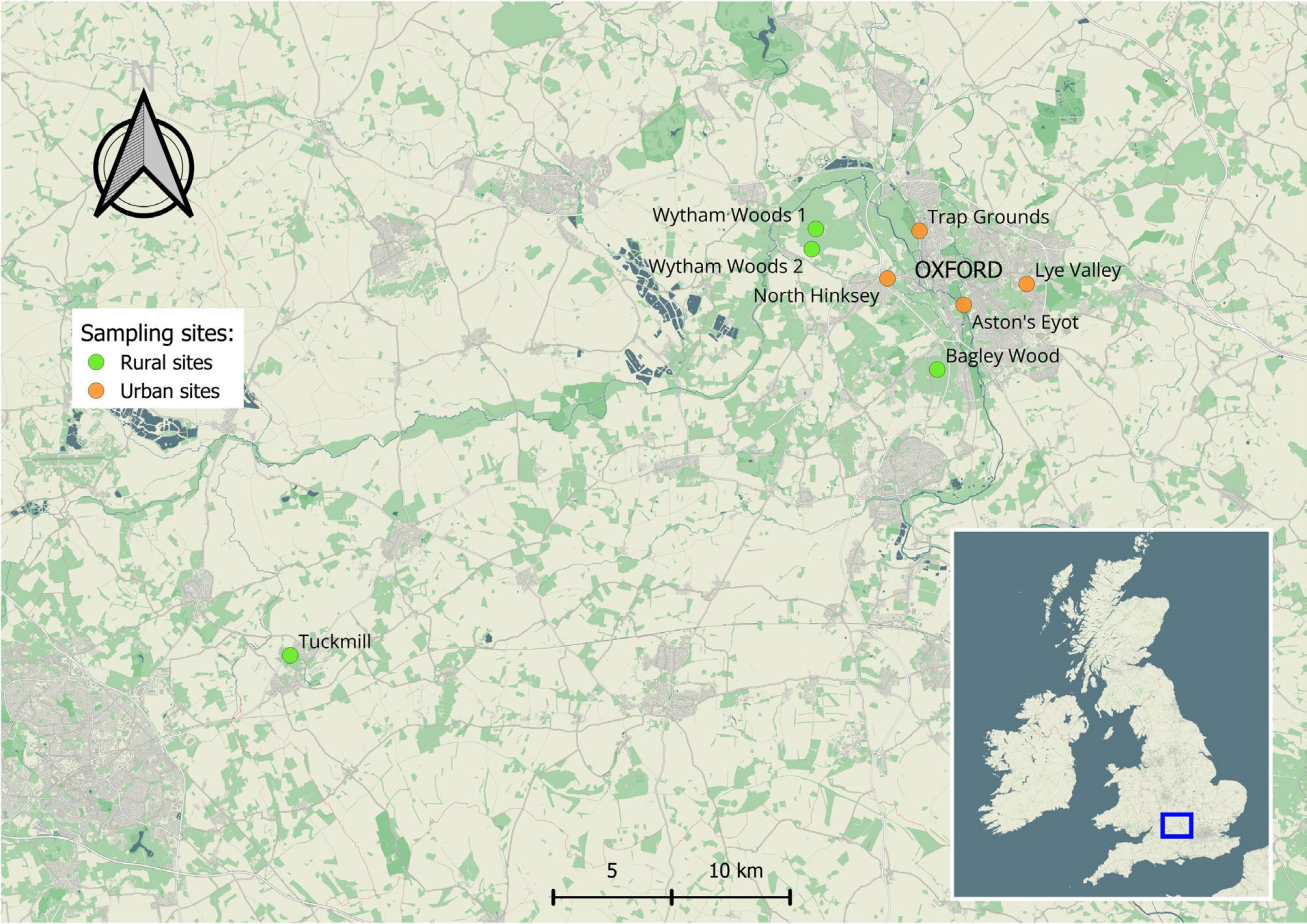
The eight sampling sites of the project. Map created using QGIS 3.28.10 with a CartoDB Positron basemap.

Sampling activities were conducted between June 13th and 22nd, 2024, across eight sites in seven parks and nature reserves in Oxfordshire, U.K. (Figure 1). Four sites were chosen in Oxford's urban areas: Trap Grounds (51.769 N, 1.272 W; ~3.8 ha), Aston's Eyot (51.741 N, 1.246 W; ~13 ha), Lye Valley (51.749 N, 1.207 W; ~4.5 ha) and North Hinksey Nature Reserve (51.751 N, 1.292 W; ~0.4 ha); the other four were chosen in rural areas: Wytham Woods (51.770 N, 1.336 W; 51.762 N, 1.338 W; ~423.8 ha, a large and intensively studied nature reserve in which we sampled two separate sites), Tuckmill Nature Reserve (51.609 N, 1.655 W; ~5.7 ha) and Bagley Wood (51.717 N, 1.262 W; ~230 ha). All sites were deliberately chosen to include woodland habitats, in order to ensure a consistent habitat type for the sampling activities across both urban and rural locations. We focussed on the British snail forest fauna in this study as it is the most diverse, and includes many species adapted to disturbed environments (Cameron 2008), allowing meaningful comparisons across urban and rural sites. Sampling permissions were obtained for each park and nature reserve.

### 2.2 Sampling Activities

Eight random points were generated on the map of each sampling site using ChatGPT v. 4 (OpenAI 2024), representing the positions of the sampling quadrats. Quadrats were deployed as close as possible to these points, with adjustments made in areas with obstacles or dense nettles. ChatGPT was preferred over more specialised cartography software because it could be easily used in the field, and obstacles or limitations imposed by the site managers often required adjustments on site. The exact coordinates of the quadrats were recorded using Google Maps.

Each quadrat, measuring 4 m x 4 m, was delimited with a white string and was thoroughly searched for land snails using durable rubber gloves. Rocks and decaying wood were gently overturned, leaves were moved and all surfaces were carefully explored. Each quadrat was searched for 12 to 15 min, with time varying due to differences in terrain complexity given by the presence of rocks, wood, moss and garbage.

Specimens were collected in a small plastic box for subsequent sorting, identification and counting phases. After these steps, specimens were released close to their collection point. Some specimens or shells of dead specimens with remnants of tissues (depending on the authorisation received by the site managers) were collected in pure ethanol for subsequent molecular analyses.

### 2.3 Species Identification and Specimen Count

After sampling, specimens were sorted into taxonomic groups and identified following the key to UK land snails by Cameron (2008). Key identification characters included shell shape, colour and size, as well as the colour, texture and odour of the animal's soft body. Additional references were consulted to understand if the distribution and habitat preferences of the species were consistent with the identifications provided (Gregory and Campbell 2000; Gregory 2000, 2002, 2013).

**FIGURE 2:**
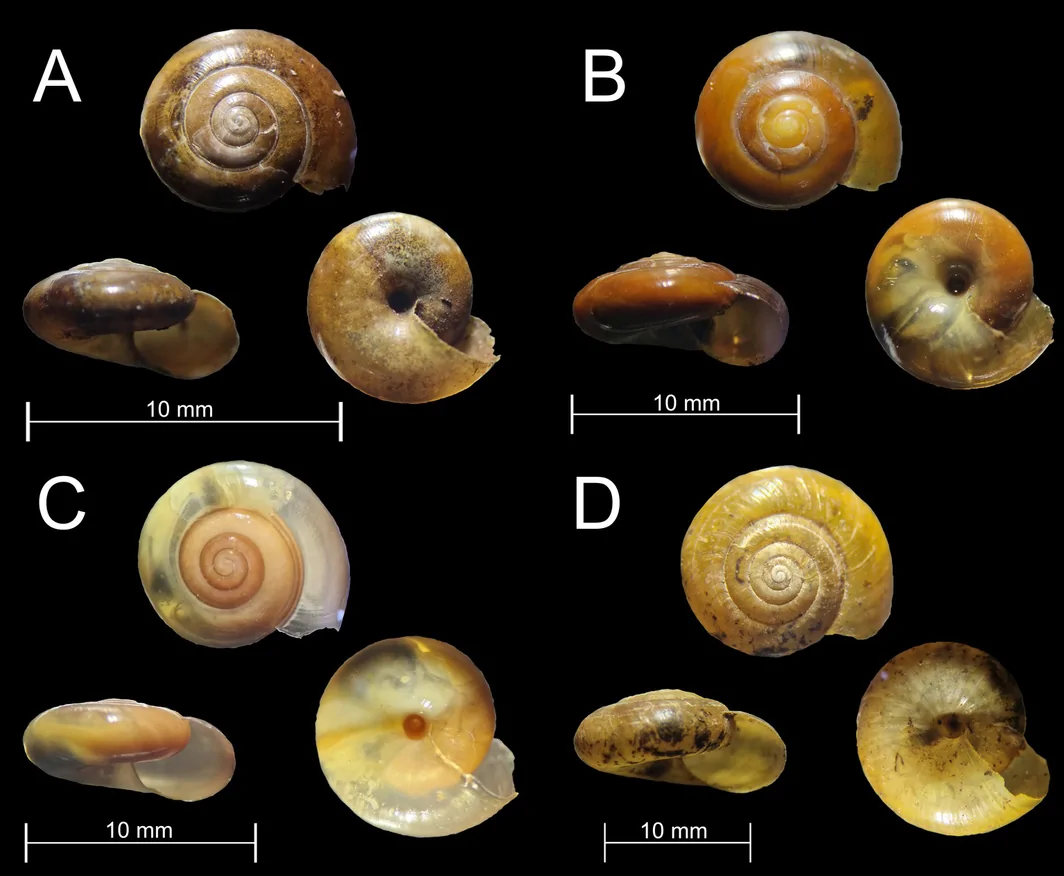
The four *Oxychilus* species found during the sampling activities. (A) The native *O. alliarius*, (B) the alien O. *navarricus*, (C) the native *O. cellarius* , and (D) the alien *O. draparnaudi*.

Specimens were identified to the lowest possible taxonomic level (usually the species level). Molecular analyses were conducted to validate the identification of most cryptic species (e.g., *Oxychilus* species, Figure 2).

The number of specimens for each taxon was counted per quadrat, including both live and dead individuals. All observable specimens were considered. Fragments of fresh but broken shells were excluded from the count as they have likely been eaten and transported by birds. Very old, broken and unidentifiable shells were excluded as well.

The shell of the largest specimen of each morphospecies was measured with a calliper for subsequent comparison with identification guides. All field data were recorded on white paper sheets to allow later corrections and annotations.

### 2.4 Molecular Analyses

The DNA of sixteen cryptic individuals was extracted using the ReliaPrep gDNA Tissue Miniprep System (Promega, USA) following the manufacturer's protocol, and the cytochrome oxidase subunit I was amplified using the primers CHACWAAYCATAAAGATATYGG (LCO1490‐JJ) and AWACTTCVGGRTGVCCAAARAATCA (HCO2198‐JJ) (Astrin and Stüben 2008). PCR cycles were set as follows: 2 min denaturation at 95°C, followed by 35 cycles of 50 s at 95°C, 50 s at 50°C and 1 min at 72°C, and a final extension of 5 min at 72°C. 3 μL of the amplification product were checked on agarose gel electrophoresis. Amplicons were then purified and sequenced by an external company (Genechron, Rome, Italy). Sequences were checked using MEGA v. 11 and blasted on GenBank and Bold Systems.

DNA was extracted and sequenced only for cryptic taxa. Notably, this analysis was conducted on the species identified morphologically as *O. cellarius* , *O. draparnaudi* , *O. navarricus* (two specimens), *T. striolatus* (two specimens), *T. hispidus* (four specimens), *Aegopinella nitidula* (two specimens), *Ashfordia granulata*, *C. lubrica* , *Vitrea* sp. and *Oxyloma elegans*.

### 2.5 Distance From Human Infrastructures and Water Sources

Three distance variables were estimated using Google Maps. Distances were measured as straight‐line Euclidean distances from the centre of each quadrat to the nearest edge of the relevant feature:

– The closest large water source (including streams, rivers, ponds and lakes):
– The closest asphalted road or parking area regularly used by cars:
– The closest human infrastructure (including buildings, rails, asphalted areas, or any other human construction, excluding paths and dirt roads not travelled by cars)

### 2.6 Statistical Analyses

All statistical analyses were conducted using R 4.3.2 (R core team, Vienna, Austria) with the Rstudio interface (RStudio Team, Boston, MA, USA).

To analyse the factors influencing the proportion of alien species and specimens in each quadrat, we fitted two binomial generalised linear models using the *glm*() function in R. The response variables were modelled using the corresponding binomial count formulation to account for the total number of species or specimens recorded in each quadrat. Independent variables included distances from roads, water sources and human structures, the urban/rural status, the area of the site (retrieved from the park/nature reserve websites), the number of native species, and several interaction terms. Interaction terms were included to test whether the effects of local environmental variables differed between urban and rural sites, and whether the effects of native diversity depended on the integrity of the nature reserve. Final models were obtained by stepwise simplification of unsupported terms, preserving model hierarchy and ecological interpretability. The significance of model terms was assessed using Wald chi‐square tests, using the *Anova*() function of the R library ‘*car*’. Final models were simplified by removing unsupported interaction terms. Multicollinearity among predictors in the final models was assessed using variance inflation factors calculated with the *check_collinearity*() function from the R package *performance*.

Since quadrats were nested within sites, we also refitted the final models as generalised linear mixed models using the *glmer*() function from the R package *lme4*, with Site included as a random intercept. However, the models showed that there was no residual variation associated with the random site variable, indicating that no residual site‐level variation remained after accounting for the fixed effects. These mixed models were used as sensitivity checks to assess whether accounting for site‐level clustering altered the results.

To further explore differences in overall community composition among sites, we performed a non‐metric multidimensional scaling (NMDS) analysis based on species abundances. Bray–Curtis dissimilarities were calculated using the *metaMDS*() function in the R package *vegan*, with two dimensions and 100 random starts.

## 3 Results

### 3.1 Reported Species

A total of 23 taxa were recorded in the quadrats, representing at least 24 species (see Table A1 for details). Most taxa were identified morphologically to species level, using ‘cfr’ where ambiguities arose, as described above. However, given the high similarity between *Trochulus hispidus* and *Trochulus striolatus* (both found in our samples), we considered these two species as a unique taxon, i.e., *Trochulus* spp. Molecular analyses confirmed the identifications provided morphologically, with percentages of identity between our sequences and sequences available online ranging from 97.3 to 100%. Sequences will be deposited in GenBank as part of a future study on the species.

Among the 23 taxa, five alien species were identified (Gregory and Campbell 2000; Cameron 2008):

– The Garden snail *Cornu aspersum*: The species, known as a crop pest, can reach a considerable size of over 40 mm (up to 31 in these samples) (Gregory 2000; Cameron 2008). It was found in the four urban sites and in Tuckmill, but only with low abundance (up to five specimens per site), and only two live specimens were found (one in Aston's Eyot, another in Lye Valley).
– The Kentish snail *Monacha cantiana* : This species was found in all sites except the second one in Wytham Woods. Its overall abundance varied widely among the sites, ranging from one specimen (Bagley Wood and first sampling site in Wytham Woods) to 28 specimens (Tuckmill). Both live and dead specimens were found in four sites, while only dead specimens were found in Tuckmill and only a live one in the first site of Wytham Woods.
– The Girdled snail *Hygromia cinctella*: Seldomly reported in previous local literature, this species was found in the four urban sites and in Tuckmill, with wide variations in abundance with only one dead specimen found in Tuckmill, two dead in Aston's Eyot, 16 dead in Trap Grounds, eight dead and two alive in Lye Valley and 9 dead and 2 alive in North Hinksey.
– The Swiss glass snail *Oxychilus navarricus*: Formerly known as *O. helveticus* (from which it obtains its common name). It resembles the native *O. alliarius* (Figure 2), but can be distinguished by its larger shell, darker mantle colour and milder garlic odour (Cameron 2008). This snail was only identified in two sites: Aston's Eyot (one alive and one dead specimen) and Tuckmill (seven alive and eight dead specimens).
– Draparnaud's glass snail *Oxychilus draparnaudi* : This predatory snail is very similar to the native *O. cellarius* (Figure 2), but with a shell diameter exceeding 12 mm, growth lines more visible and the last whorl enlarging rapidly (Cameron 2008). It was only found in Lye Valley, where large shells (up to 13.6 mm) allowed a secure identification. Both live (8) and dead (20) specimens were found at the site.

### 3.2 Community Composition

Overall specimen abundance per site was 339 at Trap Grounds, 387 at Aston's Eyot, 274 at Wytham Woods 1, 210 at Tuckmill, 99 at Bagley Wood, 364 at Lye Valley, 187 at Wytham Woods 2, and 141 at North Hinksey.

**FIGURE 3:**
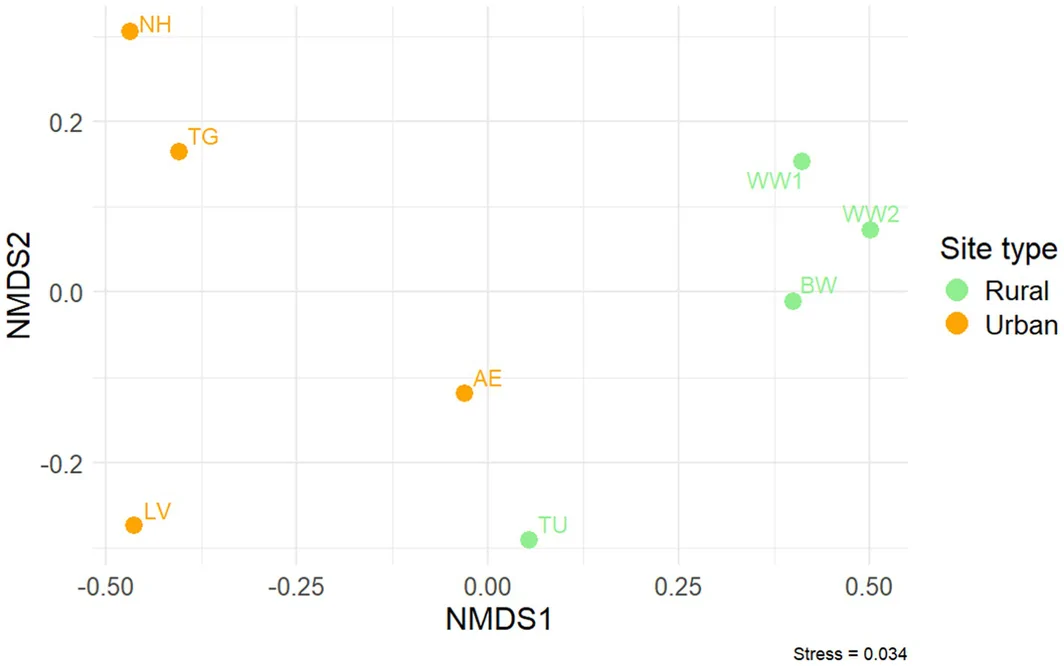
Non‐metric multidimensional scaling (NMDS) ordination based on Bray–Curtis dissimilarities calculated from site‐level species abundances. Points represent sites and colours indicate a priori urban/rural classification. Site labels are shown next to each point: Trap Grounds (TG), Aston's Eyot (AE), Wytham Woods Site 1 (WW1), Tuckmill (TU), Bagley Wood (BW), Lye Valley (LV), Wytham Woods site 2 (WW2), Noth Hinksey (NH). Stress = 0.034.

The exploratory NMDS based on Bray–Curtis dissimilarities of site‐level species abundances showed a good two‐dimensional representation of community dissimilarities, with low stress value (stress = 0.034). Most urban and rural sites are separated in the ordination space, but Tuckmill occupies a position moved towards the urban sites (Figure 3), indicating that its community composition differs from the other rural woodlands.

### 3.3 Alien Abundance and Environmental Variables

**FIGURE 4:**
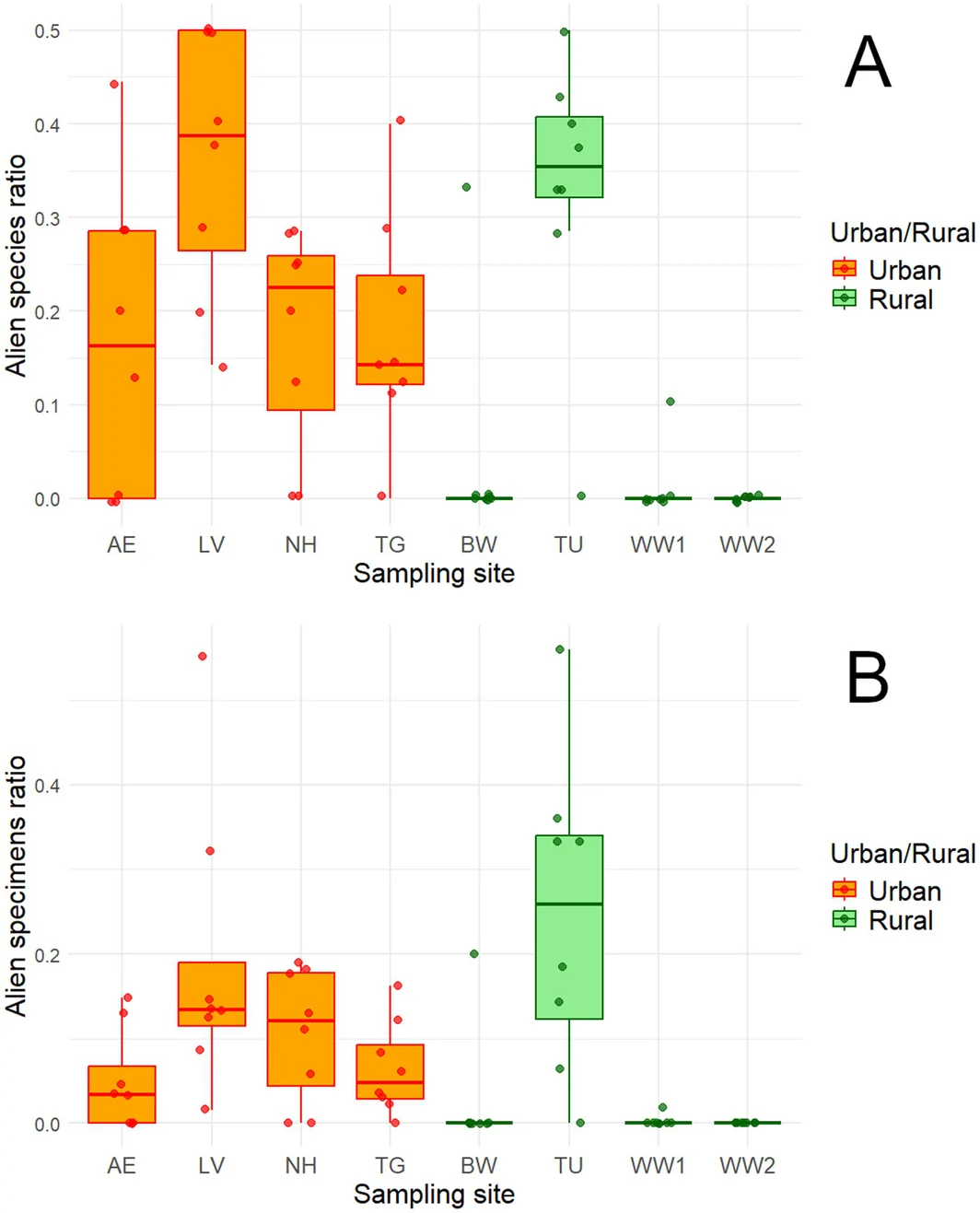
(A) The ratio of alien species to the total number of species and (B) the ratio of alien specimens to the total number of specimens across the urban (orange) and rural (green) sites: Aston's Eyot (AE), Bagley Wood (BW), Lye Valley (LV), North Hinksey (NH), Trap Grounds (TG), Tuckmill (TU), Wytham Woods sites 1 and 2 (WW1, WW2).

The two ratios of alien abundance varied sharply across the sites (Figure 4). The urban sites usually showed higher values of alien abundance, with the exception of Tuckmill.

The outputs of the two binomial models are detailed in Table 1 and Table 2.

**TABLE 1:** Output of binomial generalised linear model for the proportion of alien species.

Predictor	Estimate	SE	Likelihood ratio chisq	Df	*p*
Road distance	−0.008	0.004	4.177	1	0.041
Area	0.104	0.064	9.047	1	0.003
Urban/Rural status	0.980	0.545	0.396	1	0.529
Native species number	−0.334	0.124	5.689	1	0.017
Area:Urban/Rural status	−0.118	0.064	3.602	1	0.058
Area:Native species number	0.002	0.001	2.881	1	0.090

*Note:* The response variable is fitted as ‘cbind(NN, Total_sp – NN)’, representing the number of alien and native species recorded in each quadrat. ‘Area:Urban/Rural status’ and ‘Area:Native species number’ represent the interactions between the indicated variables. Estimates are shown on the logit scale. Values are rounded to three decimal places. The model is based on 64 quadrats, with a total of 388 species counts across quadrats used as the binomial denominator.

**TABLE 2:** Output of the binomial generalised linear model for the proportion of alien specimens.

Predictor	Estimate	SE	Likelihood ratio chisq	Df	*p*
Road distance	−0.019	0.004	22.221	1	< 0.001
Water distance	−0.005	0.004	< 0.001	1	0.985
Human structures distance	−0.006	0.006	4.652	1	0.031
Urban/Rural status	−1.592	0.851	10.906	1	< 0.001
Area	0.215	0.057	28.662	1	< 0.001
Water distance: Urban/Rural status	0.020	0.008	6.365	1	0.012
Human structures distance: Urban/Rural status	0.043	0.008	33.923	1	< 0.001
Area: Urban/Rural status	−0.251	0.057	20.142	1	< 0.001

*Note:* The response variable is fitted as ‘cbind (Abu_NN, Abu_tot – Abu_NN)’, representing the number of alien and native specimens recorded in each quadrat. ‘Water distance: Urban/Rural’, ‘Human structures distance: Urban/Rural statuses, and ‘Area: Urban/Rural status’ represent the interactions between the indicated variables. Estimates are shown on the logit scale. Values are rounded to three decimal places. The model is based on 64 quadrats, with a total of 2001 specimen counts across quadrats used as the binomial denominator.

**FIGURE 5:**
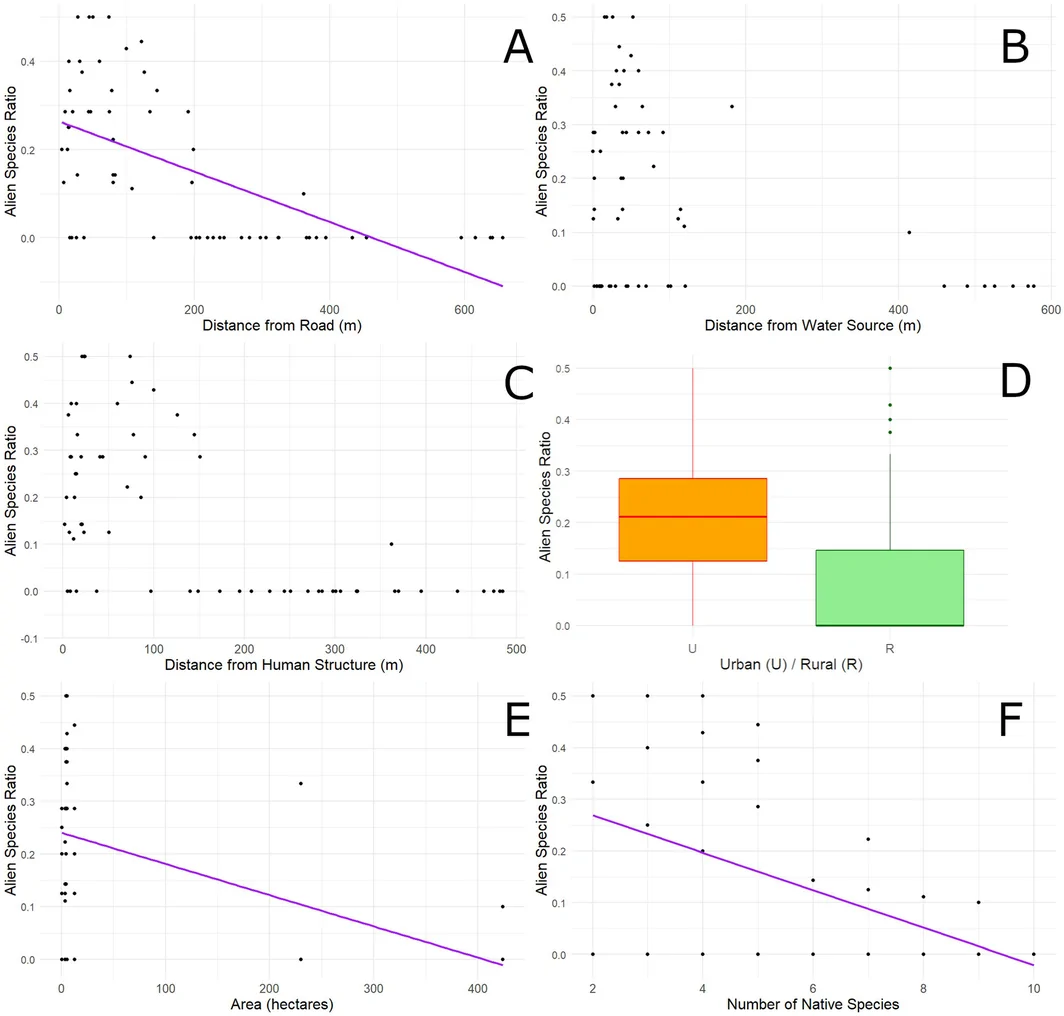
The relationship between the ratio of alien species to the total number of species and (A) the distance from roads, (B) the distance from water sources, (C) the area of the site, (D) the urban/rural status, (E) the site area, (F) the number of native species. Lines represent simple linear trend lines added for visualisation and do not correspond to fitted values from the final GLMs.

The first model (Table 1, Figure 5) indicates that the proportion of alien species is significantly influenced by the distance from roads (*p* = 0.041), the site area (*p* = 0.003) and the number of native species (*p* = 0.017). Although urban sites show a higher alien species ratio (Figure 5D), this increase is not statistically significant. The interaction between site area and urban/rural status showed marginal support (*p* = 0.058), suggesting that the effect of site area may differ between urban and rural sites. Similarly, the interaction between site area and native species richness showed weak support (*p* = 0.090), suggesting that the relationship between native richness and alien species proportion may partly depend on site size. The distance from water sources and the distance from human structures yielded no significance and were thus excluded from the model.

**FIGURE 6:**
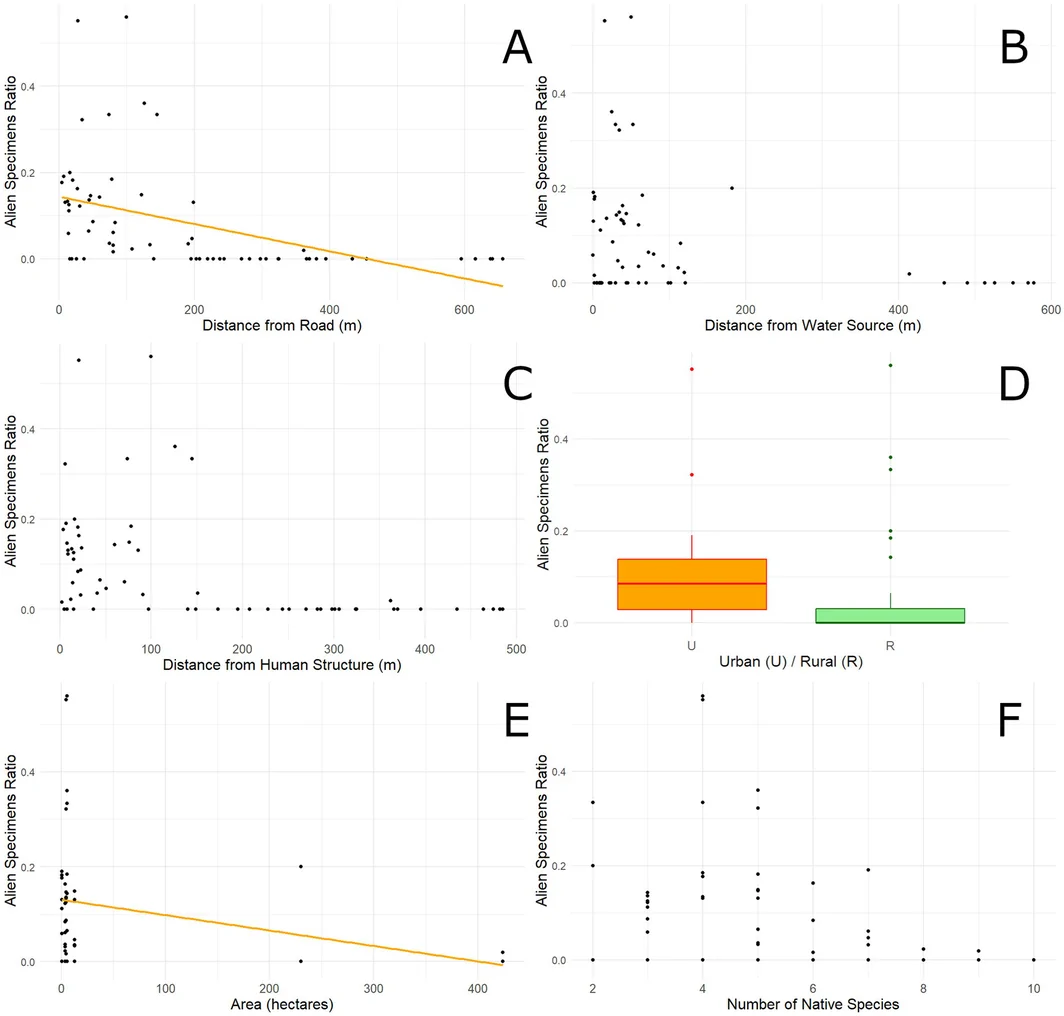
Scatterplots (A–C, E, F) and boxplot (D) indicating the relationship between the ratio of alien specimens to the total number of specimens and (A) the distance from roads, (B) the distance from water sources, (C) the area of the site, (D) the urban/rural status, (E) the site area, (F) the number of native species. Lines represent simple linear trend lines added for visualisation and do not correspond to fitted values from the final GLMs.

The second model (Table 2, Figure 6) evidences that the proportion of alien specimens is significantly influenced by the road distance (*p* < 0.001), the urban/rural status (*p* < 0.001), the site area (*p* < 0.001) and the distance from human structures (*p* = 0.031). The distance from water sources was not significant as a main effect, but its interaction with urban/rural status was significant (*p* = 0.012). Significant interactions between urban/rural status and distance from human structures (*p* < 0.001) and between urban/rural status and site area (*p* < 0.001) further indicate that the effects of these environmental variables differ between urban and rural sites. The number of native species yielded no significance and was thus excluded from the model.

Multicollinearity checks indicated high raw VIF values for some predictors, although adjusted VIF values were always below 5. We therefore retained the ecologically motivated model structure but interpreted the affected terms cautiously.

## 4 Discussion

Urbanisation and habitat fragmentation are widely recognised as main drivers of biological invasions, as they create disturbed environments that favour opportunistic and alien species while often reducing the resilience of native communities (Brasher 2003; Marvier et al. 2004; Devictor et al. 2008; Spyra et al. 2015). Consistent with general invasion theory and urban ecology, and in line with our initial expectation that anthropogenic disturbance would promote invasion in this group of organisms, the results of this study show that alien snails are more abundant in smaller, more disturbed woodland reserves, particularly those closer to roads, while larger sites with richer native communities tend to host fewer alien taxa. The significant relationship between both proportions of alien abundance and the distance from roads supports previous research, which indicates that roadsides are particularly vulnerable to invasions (Dar et al. 2015). While roads can act as dispersal corridors and sources of propagule pressure, these mechanisms are mainly studied in plant species (e.g., Mortensen et al. 2009; Lemke et al. 2019). Similarly to plants (Lemke et al. 2019), roadside management, including mowing and soil transportation, could contribute to the spread of invaders along these corridors. Moreover, roads also change the structure and quality of adjacent habitats. Roadside and edge environments are often characterised by simplified vegetation, polluted soils, reduced litter continuity, altered humidity and stronger temperature fluctuations compared with less disturbed woodland interiors (Arévalo et al. 2008; Neher et al. 2013; Lejeune et al. 2026). These microhabitat changes may reduce habitat suitability for more disturbance‐sensitive native species while leaving open niches for generalist alien taxa capable of thriving in disturbed environments. To prevent the establishment of alien flora on roadsides, some authors proposed management recommendations that should be taken into account when developing roads in nature reserves, aimed at reducing disturbance and mitigating the spread of invaders. Among these, the use of original topsoil to fill in roadside ditches, the rehabilitation of roadsides with native seeds and substrates, and the monitoring of established aliens (Tyser and Worley 1992). Likewise, for land molluscs, adopting similar strategies and promoting native vegetation can probably help mitigate the spread of non‐native taxa as well. The lack of a significant relationship between the two proportions of alien abundance and the distance from general human structures might indicate that the habitat alteration caused by other structures (e.g., houses) is less impactful than the one caused by roads.

The significant (and negative) relationship between the proportion of alien species and the number of native species can further emphasise the importance of biotic resistance offered by native taxa against invasions. On the other hand, the lack of a direct relationship between the abundance of alien specimens and the number of native species likely indicates that the specimen abundance can be influenced by other factors like litter depth, humidity, reproduction and other environmental variables (as suggested by Barrientos 2000; Nunes and Santos 2012; Liu et al. 2021); moreover, the number of specimens typically has a greater variability than the number of species, reducing the statistical power to detect a significant relationship.

The relationships between the two proportions of alien abundance and the distance from water are never significant, suggesting that availability of water sources is not a key factor in the success of invaders, at least as a main effect. While the role of humidity in shaping the abundance of many snail species is well known (Čejka and Hamerlik 2009), this result probably indicates that alien species do not show differential responses to humidity compared to native counterparts. Nevertheless, in the second model, the significance of the interaction with the urban/rural status suggests that moisture‐related effects on specimens may differ between urban and rural woodland.

Similarly, the interaction between the area and the urban/rural status is significant for specimens and close to significance for species, while the interaction between the distance from human structures and the urban/rural status is significant for specimens. This indicates that the effect of the area and human structures distance on the alien abundance varies between urban and rural sites. One possible explanation is that in urban areas, proximity to human structures often increases disturbance and thus the likelihood of alien species establishment (Spyra et al. 2015), while rural areas, generally larger, exhibit different dynamics where the effect of human structures can be mitigated by larger, more continuous natural habitats (McKinney 2002). In other words, smaller urban reserves may experience greater disturbance and more severe edge effects than rural ones (Driscoll and Donovan 2004; Guerra et al. 2017). Even the interaction between the site area and the number of native species is not far from significance, suggesting that the effect of native species on the alien species proportion might also partly vary with site size. In larger, usually rural areas, native species may exert a stronger biotic resistance, possibly due to a higher habitat heterogeneity; in smaller areas, this resistance might be diminished due to the higher levels of disturbance described above. This interpretation aligns with studies suggesting that larger, less fragmented and less disturbed areas provide better conditions for native species to maintain ecosystem stability and resist invasions (Didham 2010; Wang et al. 2022).

In this context, Oxfordshire snails represent a good case study to document the role of these phenomena in shaping terrestrial communities. Despite Oxfordshire's long history in the study of the molluscan fauna, recent literature is primarily limited to the works of Steve Gregory (Gregory 2000, 2002, 2013; Gregory and Campbell 2000). The first atlas of Oxfordshire land molluscs recorded a total of 91 species (Gregory and Campbell 2000). However, this fauna is rather dynamic: anthropogenic pressures jeopardise the persistence of several species, while facilitating the spread of others (Gregory 2000; Gregory and Campbell 2000). Given the focus on woodland areas, this result validates the approach, capturing a wide diversity of species and allowing comparisons between urban and rural sites. While including other habitat types would have certainly increased species diversity (Gregory and Campbell 2000; Cameron 2008), it would have complicated comparisons due to the lack of these habitats in several sites, particularly urban ones. No particularly rare species were found, consistent with literature suggesting that rare species are often specialists with specific habitat requirements (Reinhardt et al. 2005). Indeed, targeting such snails would require alternative survey methods, such as larger and non‐random quadrats to capture a wider diversity of habitats (as in Lange and Mwinzi 2003).

The 24 species reported in this study (Table A1) represent over one third of the total snail species reported by Gregory and Campbell 2000 in Oxfordshire. However, a limitation of our sampling design is potentially overlooking very small species. About one quarter of Oxfordshire's terrestrial molluscs are believed to be less than 3 mm in size (Gregory 2000). Although the consistency of our approach across all the sites ensures the validity of these results, this size limitation may have led to the exclusion of several small taxa. Indeed, very few specimens under 3 mm were collected. This issue is intrinsic to this type of survey design, and has been noted also by other researchers (Durkan et al. 2013). Nevertheless, none of the alien species recorded in the present study are micro‐snails, and the few alien micro‐snails known from Britain (i.e., *Paralaoma servilis* and *Lucilla singleyana, see* Cameron 2008) are extremely rarely recorded and, to our knowledge, have not been reported from Oxfordshire. Therefore, any under‐detection associated with visual searches is likely to have affected primarily the smallest native taxa rather than the alien species that formed the focus of this study. The actual proportion of alien species and specimens might have thus been systematically overestimated. In this sense, the survey design may have selectively targeted the size range that includes all alien species currently known from the local fauna. To detect and identify smaller taxa, alternative sampling methods should be considered, such as litter collection, sieving and subsequent examination under a stereomicroscope (as in Forsyth et al. 2022). While alien species were not consistently larger in our dataset, two of the five detected here ( *Oxychilus draparnaudi* and *Cornu aspersum*) are large‐bodied compared to their native counterparts of the same family. Body size may therefore be an interesting trait to investigate in future research, potentially in relation to tolerance of disturbed habitats or change in dispersal ability. For example, larger snails may be more capable of climbing vegetation, artificial surfaces, or vehicles and becoming transported by humans, consistent with the ‘climbing reflex’ (Aubry et al. 2006).

Among the 23 reported taxa, five were alien species:

- *Cornu aspersum*: this ancient introduction is now very common in much of southern England (Gregory and Campbell 2000). The low abundance and the finding of only two live specimens might indicate a partial population decline. Indeed, this phenomenon is common among alien species (including other snails such as the widespread invader *Achatina fulica* ): rapid population expansions are followed by severe demographic collapses due to factors like resource depletion, competition with other invaders, or the adaptation of native diversity (Simberloff and Gibbons 2004).
- *Hygromia cinctella*: Likely the most recent colonist snail in the county: it was first reported in Devon in 1950 (Kerney 1999), but the first record in Oxfordshire dates back only to 2000, in Oxford (Gregory and Campbell 2000). The species was not found in Lye Valley by Gregory (2002), but was reported in Trap Grounds some years later (Gregory 2013). The finding of several individuals of this species in five different sites indicates a large‐scale expansion in Oxfordshire, potentially threatening native diversity. Its presence in Tuckmill, a relatively isolated reserve, might result from the transport of soil, plants, or other organic material; nevertheless, the finding of only one dead specimen at this site raises questions about the current presence of live individuals there.
-
*Monacha cantiana* : This species represents another ancient introduction to England, and it has over the last two centuries become widespread in southern England (Gregory and Campbell 2000; Cameron 2008). Therefore, its presence in almost all the sites was reasonably expected. The single specimen found in Wytham Woods aligns with literature suggesting that it rarely penetrates woodland (Gregory and Campbell 2000).
-
*Oxychilus draparnaudi* : Typically associated with human‐disturbed habitats, its presence in Lye Valley is consistent with previous literature (Gregory 2002). The species was first reported in the county in 1926, and it has since substantially expanded, colonising highly disturbed areas like gardens and churchyards, usually in the proximity of houses (Gregory and Campbell 2000). Its absence from the other sites might indicate lower levels of disturbance compared to Lye Valley, where sampling quadrats were extremely close to houses and gardens. As a predator of earthworms and other snails (Gregory and Campbell 2000; Rondelaud et al. 2006), it constitutes a direct threat to native biodiversity.
- *Oxychilus navarricus*: This species has spread widely over the last century and is now common across much of Oxfordshire (Gregory and Campbell 2000; Cameron 2008). Although previously reported in Trap Grounds by Gregory (2013), it was not found again there in the present study. While this result might indicate an insufficient sampling effort, it aligns with a recent (June 2023) snail survey conducted by the malacologist Dr. Tom Walker on site (site managers, personal communication).

Although a priori classified as rural, Tuckmill's unexpectedly high alien species ratio (likely influenced by the road proximity and the small overall area of the site) also increased the mean ratios of alien abundance in the rural sites (Figures 4, 5, 6). The alien species collected in this study are now rather widespread in southern Britain (Gregory and Campbell 2000; Cameron 2008; Gregory 2013), and thus, their reduced abundance in larger nature reserves probably reflects the inability to adapt to such environment rather than a lack of introduction. The adaptation of alien species to the habitats of introduction is very complex, involving genetic, epigenetic and ecological processes. Genetic and epigenetic adaptations are crucial, especially given the genetic bottlenecks occurring during introductions, which can result in a reduced genetic variation and thus a lower capacity to adapt (Nota et al. 2024). However, given that these species are quite widespread in the UK, and given that pulmonate gastropods often self‐fertilise and can be resistant to a reduced genetic variation (Selander and Kaufman 1973; McCracken and Selander 1980; Doums et al. 1996), the likely explanation for their limited presence in larger reserves might be mainly ecological: richer and more diverse communities can offer the so‐called ‘biotic resistance’ against biological invasions (Beaury et al. 2020). Notably, the intricate and structured trophic relationships occurring between native taxa can limit the ecological niches available for invaders. This can happen, for example, through direct predation by native predators (Giakoumi et al. 2019), or due to the consumption of the resources aliens would need to expand (Ruesink 2007). This aligns with research showing that as parks increase in size, the proportion of disturbed areas usually decreases, promoting more diversified and resilient communities (McKinney 2002).

## 5 Conclusions and Future Perspectives

Urbanisation and habitat fragmentation are key drivers of invasions, and in this context, it is essential to investigate the importance of the different sources of human pressure in shaping the structure of natural communities. This study provides new insights intdo the diversity and distribution of woodland land snails in Oxfordshire, identifying the factors that influence the presence and abundance of alien taxa. Choosing to consistently focus on woodland areas, we captured a wide range of species, allowing comparisons between the urban and rural sites. The research underscores the importance of human disturbance in facilitating biological invasions of alien land snails. The strong relationship between the abundance of invaders and the proximity to roads highlights the role of anthropogenic activities in developing conditions favourable to invaders and calls for special attention in the development of these infrastructures. However, the lack of a significant relationship between alien abundance and the distance from generic human structures suggests that not all the types of human disturbance are equally impactful. These results also evidence that urban and rural sites can respond differently to human disturbance, with urban areas being more susceptible to invasion probably due to the smaller, more fragmented habitats.

Understanding how disturbance and habitat fragmentation influence biological invasions remains challenging, particularly for understudied terrestrial invertebrates. Future research should adopt a cross‐taxonomic approach to assess whether the patterns observed here are consistent across different groups and habitat types, helping to identify general mechanisms driving invasion success and resistance in anthropised environments. Furthermore, studies detailing the ecological interactions between native and alien snail species, including competition for resources and predation, can shed light on the exact mechanisms driving biotic resistance in larger reserves. The use of other sampling methods, such as litter collection followed by microscopic analyses, is also recommended for future research on land snails or other small invertebrates; indeed, these techniques would allow capturing smaller taxa that might have been overlooked in the present study, providing a complete picture of the invertebrate biodiversity.

## Author Contributions

**Alessandro Nota:** conceptualization (lead), data curation (lead), formal analysis (lead), investigation (lead), methodology (lead), project administration (lead), validation (lead), visualization (lead), writing – original draft (lead), writing – review and editing (equal). **Thomas Hesselberg:** conceptualization (supporting), formal analysis (supporting), methodology (supporting), supervision (lead), writing – original draft (supporting), writing – review and editing (equal).

## Funding

The authors have nothing to report.

## Conflicts of Interest

The authors declare no conflicts of interest.

## Data Availability

Data and code are available from Dryad at https://doi.org/10.5061/dryad.1vhhmgr89 (private for peer review, Reviewer sharing link: http://datadryad.org/share/LINK_NOT_FOR_PUBLICATION/rdnr3IdTZhQq1KMcv64Qt6lrgq7327WSx_o9FO3LenA).

## Appendix A

**TABLE A1:** List of the taxa reported in the eight sampling sites: Trap Grounds (TG), Aston's Eyot (AE), Wytham Woods Site 1 (WW1), Tuckmill (TU), Bagley Wood (BW), Lye Valley (LV), Wytham Woods site 2 (WW2), Noth Hinksey (NH), where Y = present and *N* = absent.

	TG	AE	WW1	TU	BW	LV	WW2	NH	Total
*Aegopinella nitidula*	Y	Y	Y	Y	Y	Y	Y	Y	8
*Arianta arbustorum*	N	Y	Y	Y	Y	Y	Y	N	6
*Ashfordia granulata*	Y	N	N	N	N	Y	N	Y	3
*Cepaea hortensis*	N	Y	Y	Y	Y	Y	Y	Y	7
*Cepaea nemoralis*	Y	Y	Y	Y	Y	N	Y	Y	7
*Cernuella virgata*	N	N	N	Y	N	N	N	N	1
*Clausilia bidentata*	Y	N	Y	Y	Y	Y	Y	N	6
*Cochlicopa* cfr *lubrica*	Y	Y	Y	Y	Y	Y	Y	Y	8
*Cochlodina laminata*	N	N	Y	N	Y	N	Y	N	3
***Cornu aspersum***	Y	Y	N	Y	N	Y	N	Y	5
*Discus rotundatus*	Y	Y	Y	Y	Y	N	Y	N	6
***Hygromia cinctella***	Y	Y	N	Y	N	Y	N	Y	5
*Merdigera obscura*	Y	N	Y	N	Y	Y	N	N	4
***Monacha cantiana***	Y	Y	Y	Y	Y	Y	N	Y	7
*Oxychilus cfr alliarius*	Y	Y	Y	Y	Y	Y	Y	Y	8
*Oxychilus cfr cellarius*	Y	Y	Y	N	Y	N	Y	Y	6
***Oxychilus draparnaudi***	N	N	N	N	N	Y	N	N	1
***Oxychilus navarricus***	N	Y	N	Y	N	N	N	N	2
*Oxyloma elegans*	N	N	N	N	N	N	N	Y	1
*Pomatias elegans*	N	N	Y	N	Y	N	N	N	2
*Trochulus* spp.	Y	Y	Y	Y	Y	Y	Y	Y	8
*Vitrea* sp.	N	N	N	N	N	N	Y	N	1
*Vitrina* cfr *pellucida*	N	N	N	N	N	Y	N	N	1
Total	13	13	14	14	14	14	12	12	

*Note:* Alien species are in bold. The table also indicates the total number of sites in which each species was found and the total number of species for each site.
